# The work stress, occupational burnout, coping strategies and organizational support of elite sports coaches in Sichuan Province: the mediating role of organizational support

**DOI:** 10.3389/fpsyg.2024.1437234

**Published:** 2024-08-07

**Authors:** Liang Yu, Liang Cheng

**Affiliations:** ^1^Human Movement Science, Sichuan Sports College, Chengdu, China; ^2^Sichuan Academy of Chinese Medicine Sciences, Chengdu, China; ^3^School of Sports Medicine and Health, Chengdu Sport University, Chengdu, China

**Keywords:** wellbeing, coping strategies, stress management, athlete training, mental health

## Abstract

**Objective:**

This study investigated the relationships between job pressure, occupational burnout, organizational support and coping strategies among competitive sports coaches in Sichuan Province, China. It also assesses the impact of job pressure on occupational burnout and the mediating roles of organizational support and coping strategies.

**Methods:**

A survey was conducted with 207 competitive sports coaches from Sichuan Province, China. Basic information and data on job pressure, occupational burnout, organizational support and coping strategies were collected. Correlations between variables were analyzed, and a theoretical model for hypothesizing the mediating effects was established. A multiple regression model was used to predict the relationships between occupational burnout and job pressure, organizational support and coping strategies.

**Results:**

(1) Job pressure was significantly positively correlated with occupational burnout (*R* = 0.436, *p* < 0.001) and negative coping (*R* = 0.389, *p* < 0.001) but significantly negatively correlated with organizational support (*R* = −0.503, *p* < 0.001). Occupational burnout was significantly negatively correlated with academic title (*R* = −0.158, *p* = 0.023) and organizational support (*R* = −0.349, *p* < 0.001) but significantly positively correlated with negative coping (*R* = 0.440, *p* < 0.001). Organisational support was significantly positively correlated with positive coping (*R* = 0.222, *p* = 0.001) but significantly negatively correlated with negative coping (*R* = −0.207, *p* = 0.003). (2) Mediating effects: Job pressure indirectly affects occupational burnout via organizational support and negative coping strategies. (3) Multiple regression: Y_1_ (Job Pressure) = 69.262 + 1.172 × Emotional Exhaustion −2.231 × Emotional Support +1.041 × Negative Coping −6.554 × Academic Title (from high to low). Y_2_ (Occupational Burnout) = 25.609 + 0.141 × Job Pressure − 0.306 × Organisational Support +0.620 × Negative Coping −2.097 × Academic Title.

**Conclusion:**

Job pressure is a significant factor leading to occupational burnout among competitive sports coaches in Sichuan Province, China, and factors such as role, interpersonal relationships and career development are closely related to occupational burnout. The sense of organizational support and negative coping strategies play a mediating role between job pressure and occupational burnout. Reducing emotional exhaustion and negative coping, enhancing emotional support and improving the academic titles of coaches can help to reduce job pressure and occupational burnout among competitive sports coaches in Sichuan Province.

## 1 Introduction

Occupational burnout, an extreme manifestation of job pressure, represents the response of the body to stress (Yang et al., 2023; Altaş et al., 2024). When individuals are confronted with job pressure, they experience psychological and physical tension. Against the backdrop of China’s competitive sports system, which operates under a ‘national system’ (Cheng et al., 2022), coaches in competitive sports face numerous job pressures, particularly during the quadrennial National Games of China. This event involves competition and comparison of sports achievements across provinces, and pressure stems not only from the job itself but also from the stringent demands and high standards set by higher authorities and expectations regarding the guidance, training and competitive outcomes of athletes (Dehghansai et al., 2023; Sankey et al., 2023). Additionally, the financial income and personal advancement of coaches often depend on the competitive performance of the athletes they coach (Orunbayev, 2023). Studies have shown a strong correlation between job pressure and occupational burnout; the greater the job pressure is, the greater the likelihood that coaches will experience occupational burnout (Richards et al., 2019; Schaffran et al., 2019). A sense of organizational support plays a significant role in the relationship between job pressure and occupational burnout (Liu et al., 2021; Xu and Yang, 2021; Irfan et al., 2023). When coaches perceive greater support from the organization, they are more inclined to adopt positive coping strategies, which can mitigate the adverse effects of job pressure (Olsen et al., 2020; Potts et al., 2023). Conversely, when coaches perceive less organizational support, they may resort to negative coping strategies, which can worsen their negative emotional state and intensify job pressure (Norris et al., 2017; Lee and Chelladurai, 2018).

To the best of our knowledge, only two studies have reported on the relationship between job pressure and occupational burnout among Chinese competitive sports coaches (Mellor et al., 2015; Men et al., 2015). A survey conducted in 2015 of 302 coaches across 10 cities in China revealed that the overall condition of occupational burnout among Chinese coaches was greater than moderate, with significant manifestations of emotional exhaustion and a lack of achievement (Men et al., 2015). A measurement of 605 Chinese coaches in 2020 revealed that job pressure among coaches is generally high and that the issue of occupational burnout is severe (Mellor et al., 2015). However, the aforementioned studies did not provide appropriate improvement strategies. The primary reason is the complexity of the relationship between job pressure and occupational burnout, which necessitates an in-depth examination and verification of mediating and moderating variables (Baquero, 2023; Jeong et al., 2024). The factors that influence the relationship between job pressure and occupational burnout among coaches must be explored to effectively prevent and control occupational burnout in stress management, which can assist coaches better cope with job pressure.

Sichuan Province is not only a populous province, with a permanent population of 83.675 million people in 2024, ranking fifth in China; it is also an important province for competitive sports, with its competitive sports achievements ranking sixth nationwide. However, coaches in Sichuan are under considerable job pressure. With the aim of addressing this issue, we conducted a field survey of 207 competitive sports coaches in Sichuan Province.

The purpose of this study is to propose effective solutions to alleviate job pressure and occupational burnout among coaches. The results have significant theoretical and practical implications for enhancing the wellbeing of coaches, optimizing the management of sports organizations, improving the training outcomes of athletes and promoting the healthy development of the sports industry. Building on prior studies that have established a correlation between job pressure and occupational burnout, our hypotheses explore the nuanced relationships between these variables and the role of organizational support and coping strategies. The study hypotheses are as follows: H1: job pressure positively affects occupational burnout; H2: organizational support negatively moderates the relationship between job pressure and occupational burnout; H3: negative coping strategies positively moderate the relationship between job pressure and occupational burnout; and H4: academic titles are negatively correlated with occupational burnout.

## 2 Materials and methods

### 2.1 Participants

This study involving humans were approved by the study was conducted according to the guidelines of the Declaration of Helsinki, and approved by the Ethics Committee of the Sichuan Sports College (2023.12). All the participants have signed an informed consent form in all participants. As of April 2024, Sichuan Province has a total of 331 competitive sports coaches: 30 senior coaches, 96 associate senior coaches, 103 intermediate coaches and 102 junior coaches. We conducted a field survey via random sampling, and 207 participants were ultimately included in the study, resulting in an effective survey response rate of 62.5% (Table 1). These coaches come from 21 prefecture-level cities and states in Sichuan Province and cover 15 Olympic sports categories (including track and field, basketball, volleyball, and football), suggesting a broad demographic representation. Among the surveyed coaches, 149 are males and 58 are females. The average age is 41.1 ± 5.5 years, and the average coaching tenure is 13.7 ± 6.5 years. In terms of length of tenure, 32 coaches have 5 years or less of experience (15.5%), 59 coaches have 6–10 years of experience (28.5%), 47 coaches have 11–15 years of experience (22.7%), 31 coaches have 16–20 years of experience (15.0%), and 38 coaches have 21 years or more of experience (18.4%).

**Table 1:** Basic information of the study participants.

Indicator	Male	Female	Total	*p*
*N*	149	58	207	
Age (years)	41.7 ± 9.5	39.7 ± 8.0	41.1 ± 5.5	0.169
Height (cm)	167.2 ± 5.6	177.3 ± 6.2	172.3 ± 6.7	0.012
Weight (kg)	63.6 ± 9.2	76.5 ± 8.6	70.2 ± 11.3	<0.01
Coaching experience (years)	14.5 ± 8.3	11.8 ± 7.3	13.7 ± 6.5	0.031
5 years and below (*n*, %)	20 (13.4%)	12 (20.7%)	32 (15.5%)	
6–10 years (*n*, %)	39 (26.2%)	20 (34.5%)	59 (28.5%)	
11–15 years (*n*, %)	33 (22.1%)	14 (24.1%)	47 (22.7%)	
16–20 years (*n*, %)	27 (18.1%)	4 (6.9%)	31 (15.0%)	
21 years and above (*n*, %)	30 (20.1%)	8 (13.8%)	38 (18.4%)	
Junior coach	52 (34.9%)	15 (25.9%)	67 (32.4%)	
Intermediate coach	56 (37.6%)	24 (41.3%)	80 (38.6%)	
Associate senior coach	31 (20.8%)	14 (24.1%)	45 (21.7%)	
Senior coach	10 (6.7%)	5 (8.6%)	15 (7.2%)	

In terms of the distribution of academic titles, 67 are junior coaches (32.4%), 80 are intermediate coaches (38.6%), 45 are associate senior coaches (21.7%), and 15 are senior coaches (7.2%).

### 2.2 Questionnaire survey

This study employed questionnaires tailored to the Chinese coaching population to accurately measure the job pressure, occupational burnout, perceived organizational support and coping strategies of coaches.

#### 2.2.1 Job pressure

The Occupational Stress Indicator, designed by Cooper et al. (1988) and consisting of 41 items, was revised to suit the Chinese coaching population (Mellor et al., 2015). Each item was rated on a five-point scale ranging from ‘completely disagree’ to ‘completely agree’ and was scored from 0 to 4. Eight dimensions were considered: job condition (items 1–4), role (items 5–9), interpersonal relationships (items 10–17), career development (items 18–20), job tasks (items 21–27), management affairs (items 28–34), personal accomplishment (items 35–39) and external competition (items 40 and 41). Higher scores indicate greater perceived job pressure by the coaches. Specified for the Chinese coaching population, our Cronbach’s *α* coefficient for this questionnaire was 0.868, with a composite reliability of 0.870 (Mellor et al., 2015).

#### 2.2.2 Occupational burnout questionnaire

A Chinese version of the Burnout Questionnaire revised by Guo and Xu (2017) from Maslach’s questionnaire (Schutte et al., 2000) consisting of 15 items was rated on a seven-point scale. The questionnaire included three dimensions: emotional exhaustion (items 1–5), reduced efficacy (items 6–11) and depersonalisation (items 12–15). The emotional exhaustion and depersonalisation dimensions were scored in the range of 0 to 6, whereas the reduced efficacy dimension was scored in reverse from 6 to 0. Higher scores indicate a greater degree of occupational burnout. Specified for the Chinese coaching population, our Cronbach’s *α* coefficient for this questionnaire was 0.892, with a composite reliability of 0.881 (Men et al., 2015).

#### 2.2.3 Perceived organizational support scale

The scale, developed by American psychologist Eisenberger et al. (1986) and consisting of 12 items, was rated on a five-point scale ranging from ‘very inconsistent’ to ‘fully consistent’ and scored from 0 to 4. Four dimensions were considered: systemic support (items 1–3), emotional support (items 4–6), instrumental support (items 7–9), and peer support (items 10–12). Higher scores indicate a greater perceived level of organizational support by the coaches. Specified for the Chinese coaching population, our Cronbach’s *α* coefficient for this questionnaire was 0.895, with a composite reliability of 0.821 (Mellor et al., 2015).

#### 2.2.4 Coping strategy scale

This simplified coping strategy questionnaire (Zhao et al., 2022) tailored for the Chinese population included 20 items, each rated on a four-point scale ranging from ‘never adopted’ to ‘frequently adopted’ and scored from 0 to 3. The questionnaire includes two dimensions: positive coping (items 1–12) and negative coping (items 13–20). Higher scores in the positive coping dimension indicate greater use of positive coping strategies, whereas higher scores in the negative coping dimension indicate greater use of negative coping strategies. Specified for the Chinese coaching population, our Cronbach’s *α* coefficient for this questionnaire was 0.901, with a composite reliability of 0.866 (Mellor et al., 2015).

### 2.3 Psychometric assessment of the questionnaire

Cronbach’s *α* was used to test the reliability of the 207 valid questionnaires. An *α* value of ≥0.70 indicates high reliability, 0.35 ≤ *α* < 0.70 is acceptable, and *α* < 0.35 indicates low reliability (Cheng et al., 2023). The reliability test showed an *α* of 0.780. In terms of assessing the test–retest reliability, a random sample of 20% of the participants was retested after 7 days. The Pearson’s correlation coefficient for each indicator was *r* ≥ 0.812, indicating a high degree of reliability in the questionnaire.

### 2.4 Statistical analysis

Statistical analyses were performed using SPSS version 20 and the SPSS macro PROCESS. All variables were initially tested for a normal distribution (Cheng et al., 2024). For normally distributed data, *t*-test was used to compare parameters between male and female participants; otherwise, the Mann–Whitney *U*-test was applied. Then, Spearman correlation analysis was used to examine the correlations among the variables. Hayes’ macro PROCESS in SPSS was employed to test the hypotheses of the theoretical model, and the bootstrapping method was used to estimate the standard errors of simple effects. In the case of testing indirect effects, a 95% bootstrap confidence interval that does not include zero indicates the presence of mediation. Finally, multiple regression models were constructed with either occupational burnout or job pressure as the dependent variables while controlling for confounding factors such as age, gender, years of vocational teaching and academic title to predict the relationships between occupational burnout or job pressure and other factors. The significance level was set at *α* = 0.05.

## 3 Results

Initially, the measured data were normally distributed. Comparisons of parameters between male and female participants were conducted via t-tests, and no statistically significant differences in the indices were detected (*p* > 0.05) (Table 2). The results of the Spearman correlation analysis are shown in Table 3 and Supplementary Table S1.

**Table 2:** Statistical results of participants’ job pressure, occupational burnout, organizational support, and coping strategies.

Indicator	Male	Female	Total	Gender comparison (*p*-value)
Job pressure (score)	94.72 ± 23.55	92.79 ± 17.43	94.18 ± 22.03	0.573
Job condition	13.54 ± 3.28	13.78 ± 2.15	13.60 ± 3.01	0.610
Role	5.07 ± 4.46	4.88 ± 3.41	5.02 ± 4.20	0.739
Interpersonal relationships	13.26 ± 6.89	12.38 ± 6.35	13.01 ± 6.75	0.386
Career development	6.40 ± 3.72	6.38 ± 3.32	6.39 ± 3.61	0.975
Job tasks	22.41 ± 5.18	23.24 ± 3.64	22.64 ± 4.81	0.266
Management affairs	18.40 ± 4.55	17.62 ± 3.58	18.18 ± 4.32	0.248
Personal achievement	10.64 ± 3.79	9.74 ± 2.87	10.39 ± 3.58	0.104
External competition	5.01 ± 2.05	4.78 ± 1.64	4.94 ± 1.95	0.447
Occupational burnout (score)	27.54 ± 11.39	28.86 ± 10.26	27.91 ± 11.10	0.445
Emotional exhaustion	14.63 ± 6.44	15.57 ± 5.18	14.89 ± 6.12	0.281
Reduced efficacy	5.32 ± 4.42	5.88 ± 2.26	5.48 ± 4.68	0.444
Alienation	7.59 ± 5.33	7.41 ± 4.53	7.54 ± 5.12	0.825
Perceived organizational support (score)	33.01 ± 9.51	33.78 ± 7.93	33.23 ± 9.10	0.590
Systemic support	8.30 ± 2.64	8.43 ± 2.07	8.33 ± 2.50	0.727
Emotional support	8.07 ± 2.90	7.95 ± 2.48	8.04 ± 2.79	0.772
Instrumental support	7.64 ± 3.13	8.07 ± 2.43	7.76 ± 2.95	0.304
Peer support	9.00 ± 2.12	9.33 ± 1.80	9.09 ± 2.04	0.302
Positive coping strategies (score)	24.41 ± 6.44	23.98 ± 6.24	24.29 ± 8.19	0.668
Negative coping strategies (score)	8.11 ± 4.92	8.40 ± 4.21	6.39 ± 4.73	0.701

**Table 3:** Correlations of job pressure, occupational burnout, organizational support, and coping strategies among participants (*N* = 207).

		Coaching tenure	Age	Academic title	Job pressure	Occupational burnout	Organisational support	Positive coping	Negative coping
Job pressure	R	0.051	−0.028	0.131		0.436	−0.503	0.075	0.389
	*p*	0.466	0.691	0.060		0.000	0.000	0.285	0.000
Occupational burnout	R	0.071	0.061	−0.158	0.436		−0.349	−0.052	0.440
	*p*	0.309	0.380	0.023	0.000		0.000	0.458	0.000
Organisational support	R	0.051	0.085	−0.121	−0.503	−0.349		0.222	−0.207
	*p*	0.470	0.226	0.082	0.000	0.000		0.001	0.003

The results of the correlation analysis are as follows: academic title (from high to low) was significantly negatively correlated with burnout total scores (*R* = −0.158, *p* = 0.023), emotional exhaustion (a subscale of burnout, *R* = −0.169, *p* = 0.015), alienation (a subscale of burnout, *R* = −0.189, *p* = 0.006) and instrumental support (a subscale of organizational support, *R* = −0.151, *p* = 0.030). Work pressure was significantly positively correlated with occupational burnout (*R* = 0.436, *p* < 0.001) and negative coping (*R* = 0.389, *p* < 0.001) but significantly negatively correlated with organizational support (*R* = −0.503, *p* < 0.001). Further correlation analysis of the eight subscales of work pressure with the three subscales of occupational burnout, negative coping and the four subscales of organizational support revealed that job security was significantly positively correlated with emotional exhaustion (*R* = 0.151, *p* = 0.030) but significantly negatively correlated with emotional support (*R* = −0.305, *p* < 0.001) and peer support (*R* = −0.184, *p* = 0.008). Role was significantly positively correlated with emotional exhaustion (*R* = 0.322, *p* < 0.001), alienation (*R* = 0.343, *p* < 0.001) and negative coping (*R* = 0.291, *p* < 0.001) but significantly negatively correlated with supervisory support (*R* = −0.408, *p* < 0.001), emotional support (*R* = −0.479, *p* < 0.001), instrumental support (*R* = −0.468, *p* < 0.001) and peer support (*R* = −0.426, *p* < 0.001). The sense of interpersonal relationships was significantly positively correlated with emotional exhaustion (*R* = 0.363, *p* < 0.001), alienation (*R* = 0.408, *p* < 0.001) and negative coping (*R* = 0.280, *p* < 0.001) but significantly negatively correlated with supervisory support (*R* = −0.425, *p* < 0.001), emotional support (*R* = −0.429, *p* < 0.001), instrumental support (*R* = −0.441, *p* < 0.001) and peer support (*R* = −0.387, *p* < 0.001). Career development was significantly positively correlated with emotional exhaustion (*R* = 0.373, *p* < 0.001), alienation (*R* = 0.359, *p* < 0.001) and negative coping (*R* = 0.295, *p* < 0.001) but significantly negatively correlated with supervisory support (*R* = −0.492, *p* < 0.001), emotional support (*R* = −0.530, *p* < 0.001), instrumental support (*R* = −0.501, *p* < 0.001) and peer support (*R* = −0.471, *p* < 0.001). The sense of job tasks was significantly positively correlated with emotional exhaustion (*R* = 0.310, *p* < 0.001) and negative coping (*R* = 0.232, *p* < 0.001) but significantly negatively correlated with supervisory support (*R* = −0.224, *p* = 0.001), emotional support (*R* = −0.247, *p* < 0.001), instrumental support (*R* = −0.302, *p* < 0.001) and peer support (*R* = −0.194, *p* = 0.005). The sense of management affairs was significantly positively correlated with emotional exhaustion (*R* = 0.166, *p* = 0.017) and negative coping (*R* = 0.240, *p* < 0.001) and showed no significant correlation with the various indicators of organizational support. Self-accomplishment was significantly positively correlated with emotional exhaustion (*R* = 0.380, *p* < 0.001), alienation (*R* = 0.374, *p* < 0.001) and negative coping (*R* = 0.383, *p* < 0.001) but negatively correlated with supervisory support (*R* = −0.246, *p* < 0.001). External competition was significantly positively correlated with emotional exhaustion (*R* = 0.279, *p* < 0.001), alienation (*R* = 0.247, *p* < 0.001) and negative coping (*R* = 0.332, *p* < 0.001) but significantly negatively correlated with supervisory support (*R* = −0.237, *p* < 0.001), emotional support (*R* = −0.264, *p* < 0.001), instrumental support (*R* = −0.307, *p* < 0.001) and peer support (*R* = −0.322, *p* < 0.001).

Occupational burnout was significantly negatively correlated with organizational support (*R* = −0.349, *p* < 0.001) but significantly positively correlated with negative coping (*R* = 0.440, *p* < 0.001). Further correlation analysis of the three subscales of occupational burnout with the four subscales of organizational support and negative coping revealed that emotional exhaustion was significantly negatively correlated with supervisory support (*R* = −0.270, *p* < 0.001), emotional support (*R* = −0.246, *p* < 0.001), instrumental support (*R* = −0.271, *p* < 0.001) and peer support (*R* = −0.256, *p* < 0.001) but significantly positively correlated with negative coping (*R* = 0.390, *p* < 0.001). The feeling of inefficacy was significantly negatively correlated with peer support (*R* = −0.139, *p* = 0.046). Alienation was significantly negatively correlated with supervisory support (*R* = −0.323, *p* < 0.001), emotional support (*R* = −0.235, *p* = 0.001), instrumental support (*R* = −0.224, *p* = 0.001) and peer support (*R* = −0.274, *p* < 0.001) but significantly positively correlated with negative coping (*R* = 0.398, *p* < 0.001). Organisational support was significantly positively correlated with positive coping (*R* = 0.222, *p* = 0.001) but significantly negatively correlated with negative coping (*R* = −0.207, *p* = 0.003). Further correlation analysis of the four subscales of organizational support with positive and negative coping revealed that supervisory support was significantly positively correlated with positive coping (*R* = 0.200, *p* = 0.004) but significantly negatively correlated with negative coping (*R* = −0.230, *p* = 0.001). Emotional support was significantly positively correlated with positive coping (*R* = 0.259, *p* < 0.001) but significantly negatively correlated with negative coping (*R* = −0.176, *p* = 0.011). Instrumental support was significantly positively correlated with positive coping (*R* = 0.154, *p* = 0.027) but significantly negatively correlated with negative coping (*R* = −0.146, *p* = 0.035). Peer support was significantly positively correlated with positive coping (*R* = 0.210, *p* = 0.002) but significantly negatively correlated with negative coping (*R* = −0.139, *p* = 0.045).

**Figure 1:**
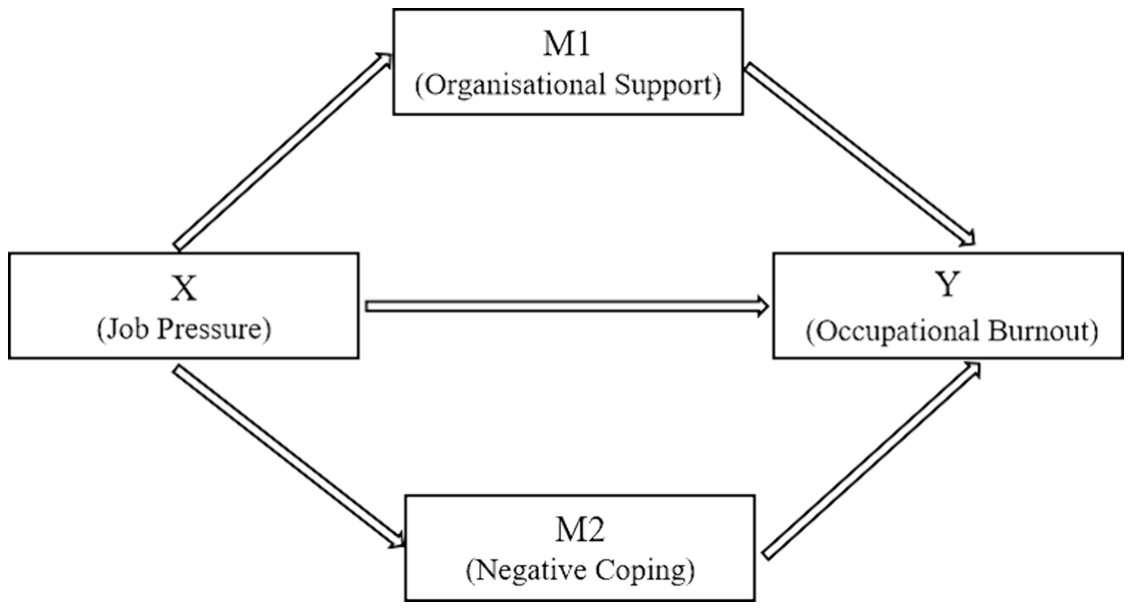
Mediation effect diagram caption.

This study examined the parallel mediating effects of organizational support and negative coping styles on the relationship between job pressure and occupational burnout (Figure 1). In terms of the total effect, the overall impact of job pressure (X) on occupational burnout (Y) was 0.2393, indicating that the influence of X on Y, without considering the mediating roles of organizational support and negative coping (M1 and M2), was significant at 0.2393 (*p* < 0.01). The direct influence of X on Y, after accounting for organizational support and negative coping, was 0.1237, which was also significant (*p* < 0.01). Finally, regarding the indirect effect (mediation effect), the mediated impact of X on Y through M1 and M2 was 0.1156, with a bootstrap standard error of 0.0262. The 95% confidence interval did not contain zero (i.e., the bootstrap lower limit confidence interval [BootLLCI] was 0.0676, and the upper limit [BootULCI] was 0.1702), indicating that the mediating effect was significant. The direct effect of X on Y was 0.2393. The indirect effect, which is the difference between the total and direct effects [0.2393–0.1237 = 0.1156], signifies that M1 and M2 mediate the relationship between X and Y, with this portion of the effect being transmitted through the mediating variables M1 and M2. These findings suggest that the impact of job pressure on occupational burnout among competitive sports coaches is moderated by organizational support and negative coping strategies.

This study constructed a multiple regression model with job pressure as the dependent variable and occupational burnout, organizational support and coping strategies as the independent variables while considering confounding factors such as age, gender, years of vocational teaching and academic title. However, a regression model could not be established. Upon further consideration of the subscales of occupational burnout, organizational support and coping strategies, the results indicated that job pressure [*F*_(4,202)_ = 38.974, *p* < 0.001] was statistically significant, with a constant value of 69.262. Multiple regression revealed relationships for emotional exhaustion (*β* = 1.172, *p* < 0.001), emotional support (*β* = −2.231, *p* < 0.001), negative coping (*β* = 1.041, *p* < 0.001) and academic title (*β* = −6.554, *p* = 0.003). The regression equation for predicting the job pressure scores of competitive sports coaches was *Y*_1_ = 69.262 + 1.172 × Emotional Exhaustion −2.231 × Emotional Support +1.041 × Negative Coping −6.554 × Academic Title.

In another model, occupational burnout were taken as the dependent variable and job pressure, and organizational support and coping strategies were taken as the independent variables, while controlling for confounding factors such as age, gender, years of vocational teaching and academic title. The results showed that occupational burnout [*F*_(4,144)_ = 24.112, *p* < 0.001] was statistically significant, with a constant value of 25.609. Moreover, multiple regression relationships for job pressure (*β* = 0.141, *p* < 0.001), organizational support (*β* = −0.306, *p* < 0.001), negative coping strategies (*β* = 0.620, *p* < 0.001) and academic title (*β* = −2.097, *p* = 0.011, where the first to fourth academic titles represent full professor, associate professor, lecturer and assistant lecturer), were detected. The regression equation for predicting the occupational burnout scores of competitive sports coaches was *Y*_2_ = 25.609 + 0.141 × Job Pressure − 0.306 × Organisational Support +0.620 × Negative Coping −2.097 × Academic Title.

## 4 Discussion

The purpose of this study was to explore the relationships between job pressure, occupational burnout, perceived organizational support and coping strategies among competitive sports coaches in Sichuan Province and to evaluate the impact of job pressure on occupational burnout, as well as the mediating roles of organizational support and coping strategies. All four research hypotheses were supported by the findings of this study.

This study confirmed Hypothesis 1: job pressure positively affects occupational burnout. The correlation analysis revealed a significant positive correlation between job pressure and occupational burnout (*R* = 0.436), indicating that the greater the job pressure is, the greater the level of occupational burnout experienced by the coaches. Furthermore, the positive standardized regression coefficient (*β* = 0.141) for job pressure in the multiple regression analysis confirmed that job pressure positively influences occupational burnout. Additionally, when all eight subdimensions of job pressure were used as independent variables and occupational burnout scores were used as the dependent variable, the model results showed that all subdimensions of job pressure were positively correlated with occupational burnout scores. This finding supports the hypothesis that job pressure positively affects occupational burnout. Moreover, the mediating effects found in this study indicate that organizational support not only directly affects occupational burnout but also plays a role in moderating the relationship between job pressure and occupational burnout. When the sense of organizational support is low, the positive correlation between job pressure and the occupational burnout of coaches intensifies. Conversely, when the sense of organizational support is high, the positive correlation between job pressure and occupational burnout is attenuated. This finding provides a new perspective for understanding and managing job pressure and occupational burnout among coaches. In practice, we should focus on enhancing the job support perceived by coaches to alleviate their job pressure and burnout, thereby improving their work efficiency and job satisfaction.

The study findings are consistent with the results of surveys conducted in 2015 on 302 competitive sports coaches (Men et al., 2015) and in 2020 on 605 competitive sports coaches (Mellor et al., 2015) in China, which revealed a significant positive correlation between job pressure and occupational burnout among coaches. The results of the current study, which investigated competitive sports coaches in Sichuan Province, China, align with the aforementioned results (Mellor et al., 2015; Men et al., 2015). We analyzed potential reasons for this correlation, including the association of several factors. Firstly, competitive sports coaches are required not only to master a vast amount of subject-specific knowledge related to their discipline but also to apply this interdisciplinary knowledge to solve various problems encountered in training (Siwik et al., 2015). This long-term commitment and effort can inevitably lead to a state of extreme fatigue. Secondly, coaches are overburdened by multiple roles in both their professional and personal lives, dealing with a multitude of complex relationships. The sustained pressure associated with these roles can lead to emotional exhaustion, physical and mental fatigue and even a sense of helplessness toward one’s work (Altfeld et al., 2018). Although job pressure among coaches can partially explain their occupational burnout, the level of burnout experienced by coaches at the same pressure level is not uniform. When coaches lack specific resources, their job demands are not fully met, or when they do not receive the expected rewards, they may experience occupational burnout (Carson et al., 2018).

This study confirmed Hypothesis 2: perceived organizational support negatively moderates the relationship between job pressure and occupational burnout. Correlation analysis indicated a significant negative correlation between job pressure and organizational support (*R* = −0.503), suggesting that as job pressure increases, perceived organizational support decreases. Additionally, the subdimensions of organizational support (systemic support, emotional support, instrumental support and peer support) were negatively correlated with the subdimensions of job pressure, demonstrating the moderating role of organizational support in the relationship between job pressure and occupational burnout. This finding is consistent with the study results of Mellor et al. (2015), who proposed that organizational support has a moderating effect on the relationship between job pressure and job burnout among coaches, with coping strategies serving as a mediating variable. Several factors may be involved based on our analysis. Firstly, organizational support can function as a buffering resource for coaches facing job pressure (Knight et al., 2015). When coaches perceive support from their organization, they feel more valued and resourceful, which can help mitigate the direct impact of job pressure on occupational burnout. According to conservation of resources theory, organizational support is a resource that can help coaches maintain a balance of resources when confronted with job pressure (Lawrence and Callan, 2011). When such support is diminished, coaches may perceive a lack of resources, thereby increasing the risk of occupational burnout. Secondly, organizational support can assist coaches in addressing job demands. Coaches who feel a high level of organizational support are likely to view job pressure as a manageable challenge rather than an insurmountable obstacle (Kristiansen and Roberts, 2010). Furthermore, organizational support may reduce the sense of emotional exhaustion among coaches, a key dimension of occupational burnout (Smollan, 2017), by providing emotional solace and practical assistance; it can also enhance the sense of achievement of coaches and reduce their feelings of alienation from their work. Finally, organizational support can indirectly alleviate job pressure by improving the work environment and conditions, such as by providing additional resources, training and career development opportunities.

The present study confirmed Hypothesis 3: negative coping strategies positively moderate the relationship between job pressure and occupational burnout. Correlation analysis revealed a significant positive correlation between job pressure and negative coping strategies (*R* = 0.389), indicating that as job pressure increases, coaches are likely to adopt negative coping strategies. Additionally, negative coping was significantly positively correlated with all eight subdimensions of job pressure, further supporting Hypothesis 3. Moreover, the positive standardized regression coefficient (*β* = 0.620) for negative coping strategies in the multiple regression analysis further illustrates that these strategies intensify the impact of job pressure on occupational burnout. We analyzed potential reasons that may be associated with the following factors. Firstly, when coaches face job pressure, they may employ various coping strategies. Negative coping strategies, such as avoidance, self-blame or emotional venting, may provide short-term relief from stress but can exacerbate occupational burnout in the long term (Edú et al., 2022). Secondly, the cumulative effect of negative coping can increase the accumulation of psychological stress, thereby intensifying the emotional exhaustion and reduced sense of achievement associated with occupational burnout (Bakker and Vries, 2021). Furthermore, negative coping may deplete the psychological resources of an individual, as these strategies typically do not effectively resolve issues and may instead impose additional psychological burdens (Hershcovis et al., 2018). Coaches who resort to negative coping strategies may struggle with emotional regulation, hindering their recovery from job pressure and increasing their sense of burnout. Additionally, negative coping strategies may affect the satisfaction with the work environment of coaches, as they may feel incapable of effectively dealing with workplace challenges (Cho et al., 2021). Negative coping strategies can also reduce the social support coaches receive from colleagues or the organization, as these strategies may hinder effective communication and collaboration. The prolonged use of negative coping strategies may lower the self-efficacy of coaches, leading them to doubt their abilities, thus increasing the risk of occupational burnout. As a way of mitigating occupational burnout, sports organizations should consider providing training in coping strategies and psychological support to help coaches develop more effective stress management skills.

The present study confirmed Hypothesis 4: academic title is negatively correlated with occupational burnout, indicating that the higher the academic title is, the lower the level of occupational burnout. Correlation analysis revealed a significant negative correlation between academic title and the total score of occupational burnout, as well as its subdimensions (emotional exhaustion and depersonalisation), supporting the hypothesis that academic title is negatively associated with occupational burnout. Additionally, multiple regression analysis revealed a negative correlation between academic title and occupational burnout scores (*β* = −2.097), implying that coaches with lower academic titles (junior level) are at greater risk of occupational burnout than are those with higher academic titles (senior level). This finding may be related to the greater job pressure and less resource support faced by junior coaches. To our knowledge, no studies have reported on the relationship between academic titles and occupational burnout among competitive sports coaches in China, and our findings provide a basis for similar future research. We analyzed potential reasons that may be associated with the following factors. Firstly, junior coaches are in the early stages of their career development, a phase that is typically accompanied by greater uncertainty and challenges. Coaches need to invest more time and effort to enhance their coaching skills and professional status (Müller et al., 2020). Secondly, coaches with higher academic titles have greater status and greater authority within the organization, enabling them to better control their work environment and reduce job pressure. Furthermore, senior coaches who have achieved certain professional accomplishments and who have received organizational recognition experience a greater level of satisfaction, which can serve as a protective factor, reducing the risk of occupational burnout (Hill et al., 2021).

This study established multiple regression models with job pressure or occupational burnout scores as the dependent variables, taking into account confounding factors such as age, gender, years of vocational teaching and academic title, to predict the relationships between job pressure or occupational burnout and other factors. Our findings hold significant practical value for developing effective intervention measures and management strategies. The regression model for job pressure developed in this study was *Y*_1_ = 69.262 + 1.172 × Emotional Exhaustion −2.231 × Emotional Support +1.041 × Negative Coping −6.554 × Academic Title (ranging from high to low). Thus, reducing emotional exhaustion and negative coping and enhancing emotional support and the academic titles of coaches can contribute to lowering job pressure. The regression model for occupational burnout developed in this study was *Y*_2_ = 25.609 + 0.141 × Job Pressure − 0.306 × Organisational Support +0.620 × Negative Coping −2.097 × Academic Title. Therefore, increasing organizational support and the academic titles of coaches and reducing negative coping can help lower occupational burnout. We recommend that sports organizations in Sichuan Province provide further support and opportunities for career advancement to coaches.

In light of the findings, this study offers several practical implications for the field of sports coaching. Firstly, it suggests that sports organizations should prioritize enhancing organizational support to alleviate job pressure and prevent burnout among coaches (Chang et al., 2024). Secondly, the development of targeted training programs aimed at improving coping strategies could be beneficial. Lastly, the recognition of the negative impact of job pressure on occupational burnout underscores the need for policy interventions that protect the well-being of coaches and promote a healthy work environment. These implications highlight the study’s contribution to both the theoretical understanding of job pressure and burnout, as well as its practical utility in shaping sports management strategies.

## 5 Study limitations

This study has certain limitations. Firstly, although the mediation analysis showed that organizational support and negative coping styles play a mediating role between job pressure and occupational burnout, the mediating effect of positive coping strategies was not discussed. Future research may benefit from integrating positive coping strategies as a mediating variable to fully understand how coping strategies affect the relationship between job pressure and occupational burnout. Secondly, while our multiple regression model considered some confounding factors, other unidentified or uncontrolled confounding factors, such as personal values, job satisfaction and the work environment of the coaches, may be considered. Subsequent studies may focus on collecting more variables that can affect occupational burnout and controlling for them in the model. Finally, given the cross-sectional design of our study, causality could not be established. Future research will be designed as a longitudinal intervention study based on the findings of this study to better alleviate job pressure and occupational burnout among coaches.

## 6 Conclusion

Job pressure is a significant factor leading to occupational burnout among competitive sports coaches in Sichuan Province, China, and factors such as role, interpersonal relationships and career development are closely related to occupational burnout. Organisational support and negative coping strategies mediate the relationship between job pressure and occupational burnout. Reducing emotional exhaustion and negative coping and enhancing emotional support and the academic titles of coaches can help to lower job pressure and occupational burnout among competitive sports coaches in Sichuan Province.

## Data availability statement

The raw data supporting the conclusions of this article will be made available by the authors, without undue reservation.

## Ethics statement

This study involving humans were approved by the study was conducted according to the guidelines of the Declaration of Helsinki, and approved by the Ethics Committee of the Sichuan Sports College (2023.12). All the participants have signed an informed consent form in all participants. The studies were conducted in accordance with the local legislation and institutional requirements. The participants provided their written informed consent to participate in this study. Written informed consent was obtained from the individual(s) for the publication of any potentially identifiable images or data included in this article.

## Author contributions

LY: Conceptualization, Data curation, Formal analysis, Funding acquisition, Investigation, Methodology, Project administration, Resources, Software, Supervision, Validation, Visualization, Writing – original draft, Writing – review & editing. LC: Conceptualization, Data curation, Formal analysis, Funding acquisition, Investigation, Methodology, Project administration, Resources, Software, Supervision, Validation, Visualization, Writing – original draft, Writing – review & editing.
