# Supplementary material for: The work stress, occupational burnout, coping strategies and organizational support of elite sports coaches in Sichuan Province: the mediating role of organizational support
Source: Front Psychol. 2024 Aug 7;15:1437234. doi: 10.3389/fpsyg.2024.1437234 (PMC11335728; doi:10.3389/fpsyg.2024.1437234)
Supplement: Supplementary file 1 [file Data_Sheet_1.PDF]

Supplementary Table 1 Correlations between the Sub-Indicators of Participant Work Pressure, Job Burnout, Organisational Support, and Coping Strategies (N = 207)

Indicator		Coaching Tenure	Age	Academic Title	Job Condition	Role	Interpersonal Relationships	Career Development	Job Tasks	Management Affairs	Personal Achievement	External Competition	Job Pressure	Emotional Exhaustion	Reduced Efficacy	Alienation	Occupational Burnout	Systemic Support	Emotional Support	Instrumental Support	Peer Support	Perceived Organisational Support	Positive Coping Strategies	Negative Coping Strategies
Coaching Tenure	R	1.000	.746**	-.421**	0.015	-0.004	0.045	-0.018	0.078	0.089	0.053	-0.057	0.051	0.099	-0.071	0.113	0.071	0.063	0.044	0.025	0.049	0.051	0.026	-0.012
	p	.	0.000	0.000	0.835	0.950	0.518	0.792	0.262	0.204	0.445	0.417	0.466	0.156	0.311	0.105	0.309	0.370	0.531	0.718	0.482	0.470	0.707	0.868
Age	R	.746**	1.000	-.478**	0.009	-0.038	0.016	-0.072	0.002	-0.011	0.020	-0.095	-0.028	0.051	-0.066	0.108	0.061	0.064	0.053	0.066	0.096	0.085	-0.001	-0.107
	p	0.000	.	0.000	0.902	0.589	0.819	0.305	0.978	0.875	0.778	0.172	0.691	0.463	0.342	0.122	0.380	0.359	0.450	0.345	0.169	0.226	0.991	0.125
Academic Title	R	-.421**	-.478**	1.000	.149*	0.010	0.024	0.079	0.116	0.074	0.084	0.127	0.131	-.169*	0.040	-.189**	-.158*	-0.073	-0.064	-.151*	-0.116	-0.121	0.118	0.029
	p	0.000	0.000	.	0.032	0.882	0.734	0.261	0.096	0.291	0.228	0.069	0.060	0.015	0.566	0.006	0.023	0.298	0.361	0.030	0.095	0.082	0.090	0.676
Job Condition	R	0.015	0.009	.149*	1.000	.401**	.408**	.432**	.521**	.228**	.190**	.282**	.604**	.151*	0.033	0.134	.158*	-0.129	-.254**	-.305**	-.184**	-.273**	.178*	0.105
	p	0.835	0.902	0.032	.	0.000	0.000	0.000	0.000	0.001	0.006	0.000	0.000	0.030	0.640	0.055	0.023	0.063	0.000	0.000	0.008	0.000	0.010	0.131
Role	R	-0.004	-0.038	0.010	.401**	1.000	.659**	.545**	.289**	0.118	.257**	.290**	.691**	.322**	-0.009	.343**	.341**	-.408**	-.479**	-.468**	-.426**	-.505**	-0.014	.291**
	p	0.950	0.589	0.882	0.000	.	0.000	0.000	0.000	0.091	0.000	0.000	0.000	0.000	0.892	0.000	0.000	0.000	0.000	0.000	0.000	0.000	0.839	0.000
Interpersonal Relationships	R	0.045	0.016	0.024	.408**	.659**	1.000	.576**	.419**	.239**	.316**	.394**	.813**	.363**	-0.055	.408**	.378**	-.425**	-.429**	-.441**	-.387**	-.476**	-0.019	.280**
	p	0.518	0.819	0.734	0.000	0.000	.	0.000	0.000	0.001	0.000	0.000	0.000	0.000	0.430	0.000	0.000	0.000	0.000	0.000	0.000	0.000	0.783	0.000
Career Development	R	-0.018	-0.072	0.079	.432**	.545**	.576**	1.000	.514**	.184**	.253**	.481**	.751**	.373**	0.057	.359**	.420**	-.492**	-.530**	-.501**	-.471**	-.579**	-0.071	.295**
	p	0.792	0.305	0.261	0.000	0.000	0.000	.	0.000	0.008	0.000	0.000	0.000	0.000	0.419	0.000	0.000	0.000	0.000	0.000	0.000	0.000	0.306	0.000
Job Tasks	R	0.078	0.002	0.116	.521**	.289**	.419**	.514**	1.000	.342**	.287**	.343**	.679**	.310**	0.041	0.129	.256**	-.224**	-.247**	-.302**	-.194**	-.293**	.149*	.232**
	p	0.262	0.978	0.096	0.000	0.000	0.000	0.000	.	0.000	0.000	0.000	0.000	0.000	0.553	0.064	0.000	0.001	0.000	0.000	0.005	0.000	0.033	0.001
Management Affairs	R	0.089	-0.011	0.074	.228**	0.118	.239**	.184**	.342**	1.000	.452**	.373**	.505**	.166*	-0.085	0.134	0.120	0.024	0.091	0.064	0.033	0.055	.285**	.240**
	p	0.204	0.875	0.291	0.001	0.091	0.001	0.008	0.000	.	0.000	0.000	0.000	0.017	0.224	0.055	0.086	0.728	0.191	0.360	0.632	0.431	0.000	0.000
Personal Achievement	R	0.053	0.020	0.084	.190**	.257**	.316**	.253**	.287**	.452**	1.000	.385**	.544**	.380**	-0.081	.374**	.348**	-.246**	-0.090	-0.092	-0.097	-.152*	0.084	.383**
	p	0.445	0.778	0.228	0.006	0.000	0.000	0.000	0.000	0.000	.	0.000	0.000	0.000	0.246	0.000	0.000	0.000	0.196	0.185	0.164	0.028	0.227	0.000
External Competition	R	-0.057	-0.095	0.127	.282**	.290**	.394**	.481**	.343**	.373**	.385**	1.000	.600**	.279**	0.006	.247**	.284**	-.237**	-.264**	-.307**	-.322**	-.337**	0.062	.332**
	p	0.417	0.172	0.069	0.000	0.000	0.000	0.000	0.000	0.000	0.000	.	0.000	0.000	0.936	0.000	0.000	0.001	0.000	0.000	0.000	0.000	0.376	0.000
Job Pressure	R	0.051	-0.028	0.131	.604**	.691**	.813**	.751**	.679**	.505**	.544**	.600**	1.000	.436**	-0.012	.402**	.436**	-.434**	-.441**	-.455**	-.410**	-.503**	0.075	.389**
	p	0.466	0.691	0.060	0.000	0.000	0.000	0.000	0.000	0.000	0.000	0.000	.	0.000	0.860	0.000	0.000	0.000	0.000	0.000	0.000	0.000	0.285	0.000
Emotional Exhaustion	R	0.099	0.051	-.169*	.151*	.322**	.363**	.373**	.310**	.166*	.380**	.279**	.436**	1.000	-0.025	.574**	.800**	-.270**	-.246**	-.271**	-.256**	-.290**	-0.046	.390**
	p	0.156	0.463	0.015	0.030	0.000	0.000	0.000	0.000	0.017	0.000	0.000	0.000	.	0.721	0.000	0.000	0.000	0.000	0.000	0.000	0.000	0.514	0.000
Reduced Efficacy	R	-0.071	-0.066	0.040	0.033	-0.009	-0.055	0.057	0.041	-0.085	-0.081	0.006	-0.012	-0.025	1.000	0.000	.383**	-0.136	-0.118	-0.101	-.139*	-0.132	0.069	0.062
	p	0.311	0.342	0.566	0.640	0.892	0.430	0.419	0.553	0.224	0.246	0.936	0.860	0.721	.	0.995	0.000	0.050	0.091	0.149	0.046	0.058	0.324	0.371
Alienation	R	0.113	0.108	-.189**	0.134	.343**	.408**	.359**	0.129	0.134	.374**	.247**	.402**	.574**	0.000	1.000	.782**	-.323**	-.235**	-.224**	-.274**	-.295**	-.138*	.398**
	p	0.105	0.122	0.006	0.055	0.000	0.000	0.000	0.064	0.055	0.000	0.000	0.000	0.000	0.995	.	0.000	0.000	0.001	0.001	0.000	0.000	0.048	0.000
Occupational Burnout	R	0.071	0.061	-.158*	.158*	.341**	.378**	.420**	.256**	0.120	.348**	.284**	.436**	.800**	.383**	.782**	1.000	-.360**	-.301**	-.284**	-.315**	-.349**	-0.052	.440**
	p	0.309	0.380	0.023	0.023	0.000	0.000	0.000	0.000	0.086	0.000	0.000	0.000	0.000	0.000	0.000	.	0.000	0.000	0.000	0.000	0.000	0.458	0.000
Systemic Support	R	0.063	0.064	-0.073	-0.129	-.408**	-.425**	-.492**	-.224**	0.024	-.246**	-.237**	-.434**	-.270**	-0.136	-.323**	-.360**	1.000	.686**	.593**	.581**	.780**	.200**	-.230**
	p	0.370	0.359	0.298	0.063	0.000	0.000	0.000	0.001	0.728	0.000	0.001	0.000	0.000	0.050	0.000	0.000	.	0.000	0.000	0.000	0.000	0.004	0.001
Emotional Support	R	0.044	0.053	-0.064	-.254**	-.479**	-.429**	-.530**	-.247**	0.091	-0.090	-.264**	-.441**	-.246**	-0.118	-.235**	-.301**	.686**	1.000	.838**	.732**	.932**	.259**	-.176*
	p	0.531	0.450	0.361	0.000	0.000	0.000	0.000	0.000	0.191	0.196	0.000	0.000	0.000	0.091	0.001	0.000	0.000	.	0.000	0.000	0.000	0.000	0.011
Instrumental Support	R	0.025	0.066	-.151*	-.305**	-.468**	-.441**	-.501**	-.302**	0.064	-0.092	-.307**	-.455**	-.271**	-0.101	-.224**	-.284**	.593**	.838**	1.000	.751**	.913**	.154*	-.146*
	p	0.718	0.345	0.030	0.000	0.000	0.000	0.000	0.000	0.360	0.185	0.000	0.000	0.000	0.149	0.001	0.000	0.000	0.000	.	0.000	0.000	0.027	0.035
Peer Support	R	0.049	0.096	-0.116	-.184**	-.426**	-.387**	-.471**	-.194**	0.033	-0.097	-.322**	-.410**	-.256**	-.139*	-.274**	-.315**	.581**	.732**	.751**	1.000	.851**	.210**	-.139*
	p	0.482	0.169	0.095	0.008	0.000	0.000	0.000	0.005	0.632	0.164	0.000	0.000	0.000	0.046	0.000	0.000	0.000	0.000	0.000	.	0.000	0.002	0.045
Perceived Organisational Support	R	0.051	0.085	-0.121	-.273**	-.505**	-.476**	-.579**	-.293**	0.055	-.152*	-.337**	-.503**	-.290**	-0.132	-.295**	-.349**	.780**	.932**	.913**	.851**	1.000	.222**	-.207**
	p	0.470	0.226	0.082	0.000	0.000	0.000	0.000	0.000	0.431	0.028	0.000	0.000	0.000	0.058	0.000	0.000	0.000	0.000	0.000	0.000	.	0.001	0.003
Positive Coping Strategies	R	0.026	-0.001	0.118	.178*	-0.014	-0.019	-0.071	.149*	.285**	0.084	0.062	0.075	-0.046	0.069	-.138*	-0.052	.200**	.259**	.154*	.210**	.222**	1.000	.256**
	p	0.707	0.991	0.090	0.010	0.839	0.783	0.306	0.033	0.000	0.227	0.376	0.285	0.514	0.324	0.048	0.458	0.004	0.000	0.027	0.002	0.001	.	0.000
Negative Coping Strategies	R	-0.012	-0.107	0.029	0.105	.291**	.280**	.295**	.232**	.240**	.383**	.332**	.389**	.390**	0.062	.398**	.440**	-.230**	-.176*	-.146*	-.139*	-.207**	.256**	1.000
	p	0.868	0.125	0.676	0.131	0.000	0.000	0.000	0.001	0.000	0.000	0.000	0.000	0.000	0.371	0.000	0.000	0.001	0.011	0.035	0.045	0.003	0.000	.

N= 207； * p ＜ 0.05 ， ** p ＜ 0.01
